# Anesthiologist’s current infection control training program needs in Palestine: a quantitative study

**DOI:** 10.1186/2047-2994-4-S1-P89

**Published:** 2015-06-16

**Authors:** AS Abu Tayeh

**Affiliations:** 1 Medical Education, IMET2000-Pal, Ramallah, Palestinian Territory, Occupied

## Introduction

Hospital acquired infection (HAI) is a frequent and serious burden in Palestine. HAIs increase the morbidity and mortality rates and cause longer hospital stays and higher costs. Anesthetic equipments are potential vectors for HAIs and anesthiologists themselves may act as vectors for the transmission of such diseases. This is particularly important when performing invasive procedures and when there is a risk of contact with blood and other body fluids.

## Objectives

This study was conducted to explore the attitudes of anesthesia residents and specialists towards infection control (IC) standards and training in Palestine.

## Methods

A multi-centre, cross-sectional, descriptive study, using a self-administered questionnaire, was conducted in January-March 2015. Participants’ needs regarding IC training material and programs policies were examined using 48 items questionnaire. SPSS was used for data analysis.

## Results

Fifty-seven anesthesia doctors from nine governmental and private hospitals in West Bank responded to our survey. Most participants were male (93%) of them 66.7% were residents, and 29.8% were specialists. 61.4% had a postgraduate degree (master, diploma, PhD). One third of the respondents reported the absence of an infection control program in their departments. Interestingly, only one quarter of participants had infection control training inside and/or outside the hospital. 67.3% reported no access to an IC manual while 46.4% do not know about the presence of IC manual. The majority (98.2%) has indicated that a qualified IC training is needed.

## Conclusion

IC teaching programs should be urgently introduced and implemented in the Palestinian training curriculum for anesthiologists and anesthesia doctors in training. Access to an up-to-date IC training manual should be addressed to provide access to the best knowledge and practices of IC standards.

## Disclosure of interest

None declared.

